# A Novel Rare Missense Variation of the *NOD2* Gene: Evidences of Implication in Crohn’s Disease

**DOI:** 10.3390/ijms20040835

**Published:** 2019-02-15

**Authors:** Sara Frade-Proud’Hon-Clerc, Thomas Smol, Frédéric Frenois, Olivier Sand, Emmanuel Vaillant, Véronique Dhennin, Amélie Bonnefond, Philippe Froguel, Mathurin Fumery, Nathalie Guillon-Dellac, Corinne Gower-Rousseau, Francis Vasseur

**Affiliations:** 1 EA 2694—Santé Publique: épidémiologie et qualité des soins, University Lille, CHU Lille, F-59000 Lille, France; francis.vasseur@univ-lille2.fr; 2 EA 7364—RADEME—Maladies RAres du Developpement embryonnaire et du MEtabolisme, University Lille, F-59000 Lille, France; Thomas.SMOL@chru-lille.fr (T.S.); Frederic.FRENOIS@chru-lille.fr (F.F.); 3 CHU Lille, Institut de Génétique Médicale, F-59000 Lille, France; 4 CNRS UMR 8199, European Genomic Institute for Diabetes (EGID), Institut Pasteur de Lille, University of Lille, F-59000 Lille, France; Olivier.sand@cnrs.fr (O.S.); Emmanuel.Vaillant@cnrs.fr (E.V.); veronique.dhennin@cnrs.fr (V.D.); Amelie.bonnefond@cnrs.fr (A.B.); philippe.froguel@cnrs.fr (P.F.); 5 Department of Medicine, Section of Genomics of Common Disease, Imperial College London, London SW7 2AZ, UK; 6 Registre Epimad, Gastroenterology Unit, Amiens University Hospital, F-80054 Amiens, France; fumery.mathurin@chu-amiens.fr; 7 Registre Epimad, Service de Santé Publique, d’Epidémiologie, d’Economie de la Santé et de la Prévention, Maison Régionale de la Recherche Clinique, CHU Lille, F-59000 Lille, France; nathalie.guillon@chru-lille.fr (N.G.-D.); corinne.gower@chru-lille.fr (C.G.-R.); 8 Inserm, UMR 995—LIRIC, Université de Lille, F-59000 Lille, France

**Keywords:** Crohn’s disease, *NOD2* gene, variation, WES

## Abstract

The *NOD2* gene, involved in innate immune responses to bacterial peptidoglycan, has been found to be closely associated with Crohn’s Disease (CD), with an Odds Ratio ranging from 3–36. Families with three or more CD-affected members were related to a high frequency of *NOD2* gene variations, such as R702W, G908R, and 1007fs, and were reported in the EPIMAD Registry. However, some rare CD multiplex families were described without identification of common *NOD2* linked-to-disease variations. In order to identify new genetic variation(s) closely linked with CD, whole exome sequencing was performed on available subjects, comprising four patients in two generations affected with Crohn’s disease without R702W and G908R variation and three unaffected related subjects. A rare and, not yet, reported missense variation of the *NOD2* gene, N1010K, was detected and co-segregated across affected patients. In silico evaluation and modelling highlighted evidence for an adverse effect of the N1010K variation with regard to CD. Moreover, cumulative characterization of N1010K and 1007fs as a compound heterozygous state in two, more severe CD family members strongly suggests that N1010K could well be a new risk factor involved in Crohn’s disease genetic susceptibility.

## 1. Introduction

Crohn’s Disease (CD) is a chronic Inflammatory Bowel Disease (IBD) resulting from the interaction of environmental factors, including the intestinal microbiota, with host immune mechanisms in genetically-susceptible individuals [1]. The *NOD2* gene, involved in innate immune responses to bacterial peptidoglycan, is closely associated with CD with an odds ratio ranging from 3–36 and was initially identified through genetic linkage analyses [2]. Other studies previously reported and satisfactorily described the involvement of *NOD2* rare variants in the genetic Crohn’s disease predisposition [3,4]. Genome-Wide Association Studies (GWAS) have now reported more than 200 genetic susceptibility loci, only accounting for 20% of the contribution to the disease risk, suggesting that more loci remain to be discovered [5]. The EPIMAD Registry covers a large area of Northern France (six million inhabitants) and collects all incident CD cases [6,7]. Data from multiplex families defined by three or more than three first-degree relatives with CD have been recorded by EPIMAD. We previously reported that 22 multiplex families and genotyping evidenced that most cases from these multiplex families were related to high frequency of NOD2 R702W, G908R and L1007fs variations [8]. However, rare CD multiplex families did not display high frequency of R702W, G908R and L1007fs variations. Thus, in these families, a high prevalence of affected cases may rely on other major genetic susceptibility variants that remain to be determined. In order to identify new genetic variations with a major effect in CD, a Whole Exome Sequencing (WES) protocol has been initiated in a family with four CD cases among two generations. WES with intra-familial controls disclosed a new *NOD2* variation related to familial aggregation of the disease.

## 2. Results

**Figure 1:**
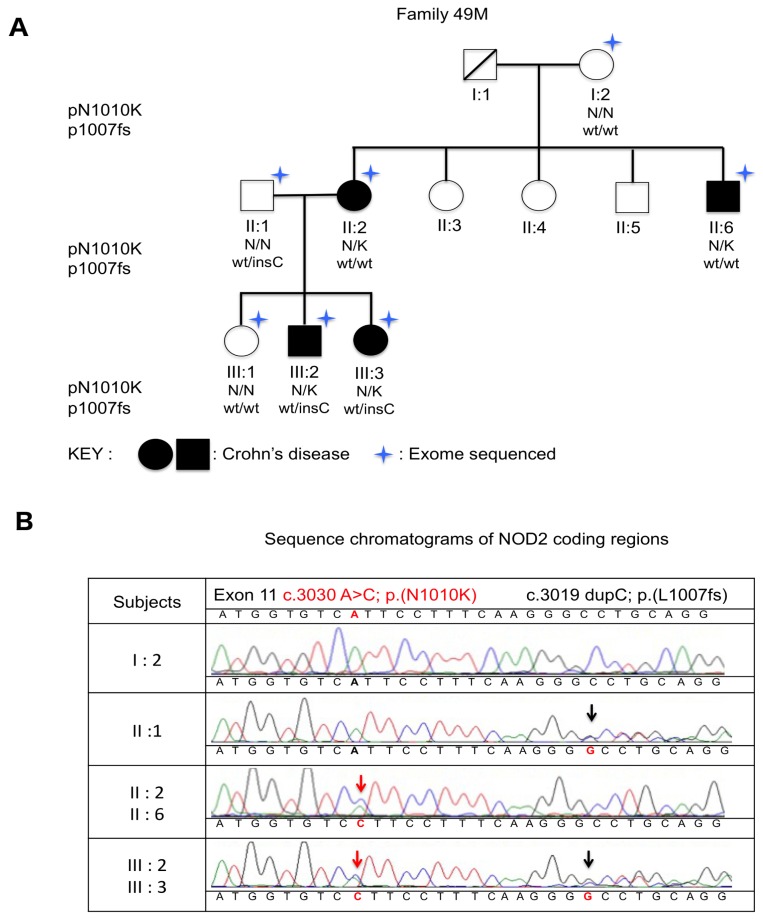
(**A**) Pedigree of family F49M with segregation of the c.3030A>C; p.(N1010K) and c.3019dupC; p.(L1007fs) variations. (**B**) Chromatograms for *NOD2* coding region in exon 11. The red arrows show the c.3030A>C nucleotide substitution consisting of the amino acid substitution N1010K. The black arrows show the c.3019dupC frameshift variation (rs2066847).

To identify new coding variations related to CD, we performed WES on multiple members of a multiplex CD family. Seven available individuals, including four patients affected with CD, were successfully whole exome sequenced. Two variations within the *NOD2* gene were identified. A novel heterozygous missense *NOD2* variation, the c.3030A>C;p.(N1010K), was identified in all affected members (Figure 1A). This variation was localized in the last exon of the *NOD2* gene. The other significant variation was the well-known c.3019dupC;p.(L1007fs), rs2066847. rs2066847 was detected in the affected members III:2 and III:3 as compound heterozygous with c.3030A>C; p.(N1010K) and was present in the unaffected father II:1 (Figure 1A). Both variations were confirmed with Sanger sequencing (Figure 1B). The *NOD2* gene was Sanger sequenced in all available subjects to ascertain the presence or the absence of c.3030A>C;p.(N1010K) and of p.L1007fs (rs2066847). Sanger sequencing allowed the authors to exclude any other *NOD2* variation.

**Figure 2:**
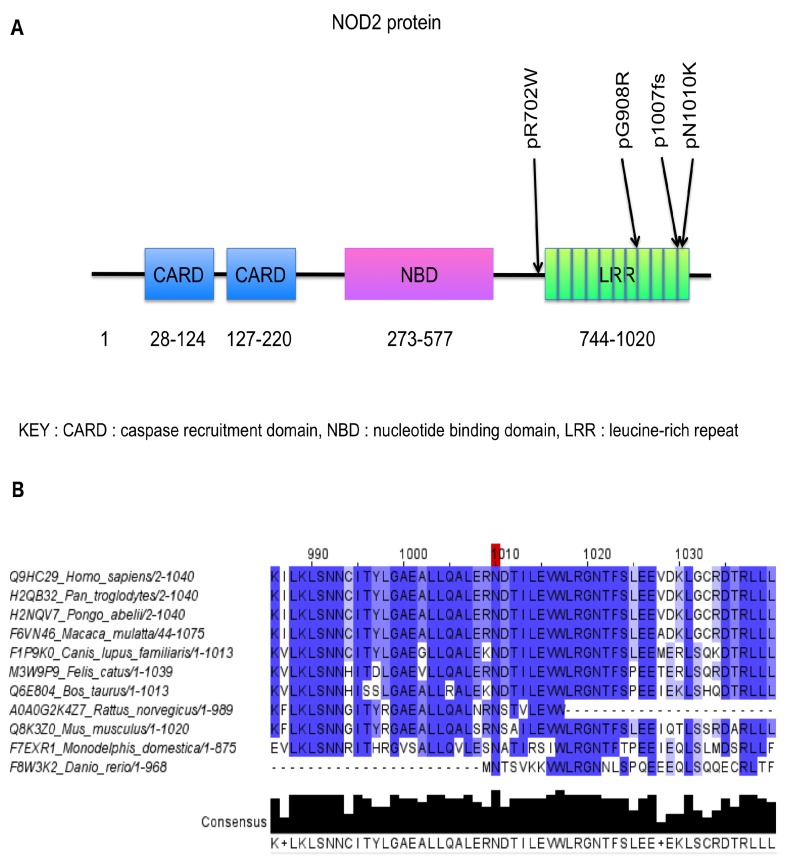
(**A**) Location of the p.R702W (rs2066844), p.G908R (rs2066845), p.L1007fs (rs2066847) and p.N1010K NOD2 protein-altering variations. (**B**) Multiple alignments for the amino acid sequence of the NOD2 proteins in 11 species in agreement with a conserved amino acid.

Like p.L1007fs, the new p.N1010K *NOD2* variation was located in the Leucine-Rich Repeat (LRR) domain of the NOD2 protein, which was already implicated in CD (Figure 2A) [9].

The heterozygous missense c.3030A>C;p.(N1010K) was absent from the public databases GnomAD, ExACand Kaviar. Coverage metrics from WES samples in public databases were considered as correct: 99.79% of control samples presented a coverage >20% for this region. This new *NOD2* variation appeared as a very rare genetic event. The deleterious effect for N1010K variation was strongly suggested by in silico predictions (Table 1).

In CADD-Phred and SIFT, the N1010K variation was predicted to be deleterious (respectively 22.6 and zero); in PolyPhen2, as possibly deleterious (0.996). The pathogenicity estimation by SIFT is based on conservation, whereas PolyPhen2 also considers available biochemical information. Although asparagine and lysine are two hydrophilic amino acids, the impact of the N1010K substitution is considered to have a significant effect according to the Grantham score (94). The multiple alignment of NOD2 protein sequence showed a high conservation level of the N1010 amino acid among Vertebrata members (Figure 2B).

**Figure 3:**
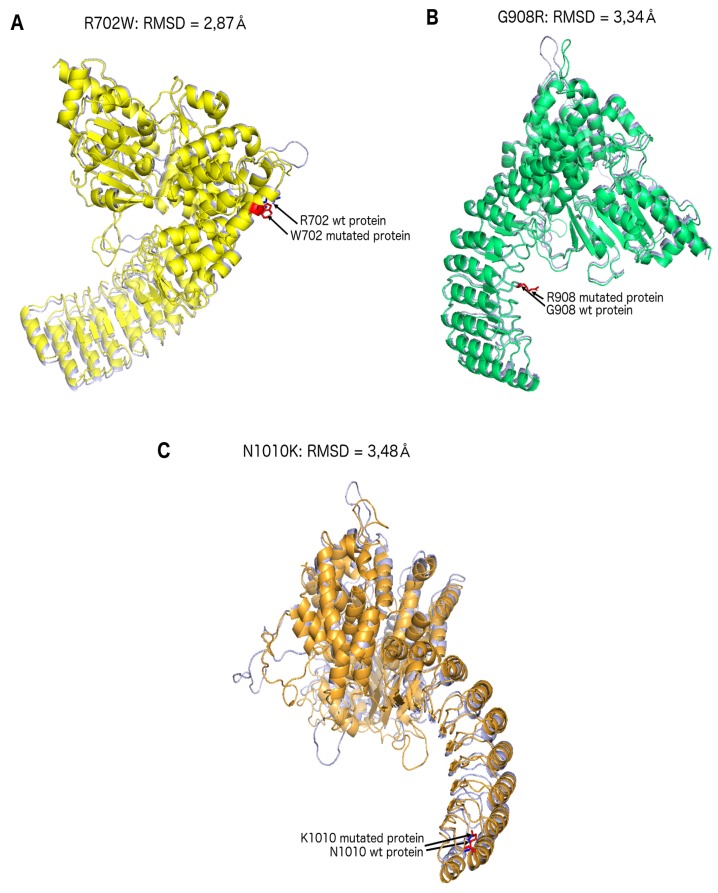
(**A**) Structural predictions of the human native NOD2 protein (amino-acids 219–1040) and the human R702W mutated NOD2 proteins (amino-acids 219–1040); (**B**) structural predictions of the human native NOD2 protein (amino-acids 219–1040) and the human G908R mutated NOD2 proteins (amino-acids 219–1040); (**C**) structural predictions of the human native NOD2 protein (amino-acids 219–1040) and the human N1010K mutated NOD2 proteins (amino-acids 219–1040).

These results were corroborated by the 3D protein modelling obtained by crystallographic simulation (Figure 3): the average displacement of each α-carbon of each amino acid was measured and made possible the quantification of the overall deformation of the protein related to the variation.

## 3. Discussion

WES study with intra-familial controls enabled a new N1010K *NOD2* variation to be detected. This may be related to familial aggregation of CD. The *NOD2* gene encodes for a protein of the NOD-Like Receptors family (NLR). This contributes to the detection of intra-cellular bacteria and their destruction, which stimulates the inflammatory response through the activation of NF-κB [10]. Three major variations altering the function of the C-terminal part of the protein have been reported as the most frequent genetic factors of CD susceptibility: p.R702W, p.G908R and p.L1007fs. However, other *NOD2* rare variations have been previously reported and were reported to be involved in CD susceptibility [3,4]. Regarding haplotypes at the *NOD2* locus in the patients III:2 and III:3 that displayed two *NOD2* variations, these two variations were located on two different haplotypes, since c.3030A>C;p.(N1010K) was maternally inherited and c.3019dupC;p.(L1007fs) paternally inherited. Thus, these patients are compound heterozygotes.

It could be speculated that the N1010K NOD2 variation impacts the NOD2 protein function. As well as the R702W, G908R and L1007fs variations, the N1010K missense is located in the Leucine-Rich Repeat (LRR) domain of the NOD2 protein. This motif is evolutionarily conserved in many proteins and associated with innate immunity [11]. The LRR domain is known to be involved in the recognition of pathogen-associated molecular patterns including components of bacteria, such as the bacterial peptidoglycan muramyl dipeptide targeting by the NOD2 protein [12]. Polymorphisms in the LRR domain are one of the most important genetic risk factors for the occurrence of CD [2,13].

The well-conserved asparagine residue involved in the N1010K variation is a polar amino acid such as lysine. However, the proximity of a conserved threonine amino acid T1012 within the NOD2 protein sequence could form a [Asn-X-Ser/Thr]motif that could be considered as a potential target for *N*-glycosylation. This could lead to a deleterious substitution [14]. Moreover, the N1010K substitution is considered to have a significant effect according to the Grantham score based on the physico-chemical difference between asparagine and lysine. Regarding the highly-conserved amino acid, the location within the functional domain of NOD2 and the absence of N1010K variation in other databases, the N1010K variation is predicted to be deleterious with CADD Phred and SIFT in silico tools, and probably deleterious with PolyPhen2. The results were similar for the two known missense variations R702W and G908R (Table 2).

Protein modelling suggests that N1010K could be associated with an alteration of the 3D structure of the human native NOD2 protein. There is no structural homology between the 3D predicted structure and the predicted hN1010K mutated NOD2 proteins, as well as for hR702W and hG908R. Crystallographic analysis previously showed that the LRR domain, between residues 745 and 1020, consisted of ten LRR units forming a horseshoe-like structure in a single curvature with alpha-helices at convex surface and beta-strand in concave faces. Thus, LRR interacts closely with the HD1 and HD2 domains through the 3D structure of NOD2 protein [15]. Maekawa and collaborators hypothesized that SNPs associated with CD located in the LRR domain would disrupt the interaction between HD1 or HD2 and the LRR domain [15]. Therefore, N1010K could disrupt or attenuate the association between the HD2 and LRR domain and could act as a loss-of-function variation. This assumption is reinforced by the significant conservation of residue N1010 and the absence of known variation in public databases.

Two out of the four patients were more severely affected than the other family members with CD according to the Montreal Classification with an earlier diagnosis [6]. Interestingly, both patients, III:2 and III:3, presented the recurrent L1007fs variation in addition to the N1010K variation in the compound heterozygous state. The L1007fs variation results in a frameshift mutation that generates a truncated NOD2 protein, which fails to co-localize to plasma membrane [9,16]. This variation resulting in a truncated protein is a major genetic risk factor of CD [2,9]. Cumulative association between the two variations in LRR domain could explain the early occurrence of CD in compound heterozygous patients III:2 and III:3. The cumulative effect of variants in a combined heterozygous state for CD was suggested by Girardelli and collaborators, but they considered variations in two different genes: K953E in *NOD2* and S159G/G351R in *IL10RA* [17]. Homozygous variations of L1007fs were identified in patients with the largest response loss to muramyl dipeptide binding by NOD2. The same effect was not reported in the case of association between L1007fs and R702W [18]. Considering proximity between L1007fs/N1010K in the LRR domain and possible loss-of-function due to N1010K, a similar impact to homozygous L1007fs could be hypothesized.

These results strongly suggest that the N1010K could be a new risk factor involved in Crohn’s disease genetic susceptibility and together with the L1007fs may explain familial aggregation of CD in the F49M family. Regarding the absence of the N1010K in all screened databases (ExAC, GnomAD, Kaviar and 1000 Genomes), it is unlikely that the N1010K rare variant should display in control subjects an MAF compatible with a successful case control study. Anyway, one could hypothesize that extensive genotyping in large groups of Crohn’s disease patients could reveal this variant in some patients. Future research should focus on discovering new genes that have implications in predicting either a better or poor overall prognosis. Functional studies should be performed to ascertain the deleterious affect of the N1010K variation.

## 4. Materials and Methods

### 4.1. Subjects

One of the 22 CD multiplex families (a family with 3 or more affected first degree relatives) from the population-based EPIMAD Registry was recruited. The L1007fs (rs2066847) mutation was present in the family, but transmitted through a non-affected husband (II:1). However, none of the R702W, G908R and L1007fs were present in affected members of Generation II (Figure 1A). In a previous study, subjects (I:2, II:2, II:6, II:1, III:2, III:3) were genotyped for the R702W, G908R and L1007fs variations of the *NOD2* gene [8]. The authors obtained an approval with a waiver of informed consent for all subjects. This work was approved by the Comité de Protection des Personnes Nord Ouest IV (the Institutional Review Board for University Hospital of Lille). The Comité de Protection des Personnes Nord Ouest IV is registered by the Office for Human Research Protections Database (IORG0009553). Four CD patients (II:2, II:6, III:2, III:3) and 3 intra-familial control subjects (I:2, II:1, III:1) covering 3 generations were recruited. Interview and clinical examination enabled Crohn’s disease to be excluded in unaffected related subjects (I:2, II:1, III:1). Moreover, for the patient II:1, there was no known family history of Crohn’s disease. The unaffected related subjects II:3, II:4 and II:5 declined to be involved in the study, but were not affected with Crohn’s disease according to the recruited subjects’ interviews. The diagnostic criteria for CD were based on clinical, radiological, endoscopic and histological findings, as described previously [6,19], and phenotypes were defined according to the Montreal Classification of CD. Age at diagnosis, clinical presentation and phenotype of CD differed between patients of this family (Table 2). This project is registered in the Clinical Trial Database: NCT02851134.

Genomic DNA was prepared from 10 mL of whole blood using the Autopure LS automate method following the manufacturers’ protocols.

### 4.2. Exome Sequencing Analysis and Computer Analyses

WES was carried out in 7 persons from the F49M family: 4 CD affected and 3 intra-familial control subjects. For this purpose, we used a NimbleGenSeqCap E2 exome v3 capture in combination with Illumina next-generation sequencing (on a HiSeq 4000 system using paired-end reads), following the manufacturers’ protocols.

Sequence reads were mapped to the human reference genome (GRCh37/hg19) using BWA v0.7.13 software. The target was covered with a mean depth of 127.7 reads in the 7 samples. Variant detection was performed with GATK v3.3 software, and candidate variants were filtered out to fit a minimum depth of 8. Variants were then annotated with the Ensembl database v75 using their Perl API, and only the non-synonymous coding, stop gain or loss, frameshift, splice site and miRNA variants were kept for further analysis. After selection of variants found only in all affected family members, the remainder were also annotated with dbSNP v135 and dbNSFP 3.0b2 (in silico functional predictions, various project allele frequencies, GO classification, expression and pathway data).

### 4.3. Sanger Sequencing Confirmation of NOD2 Variants

The presence of *NOD2* variants was confirmed by Sanger sequencing, using the standard protocol. The details regarding PCR primers and PCR conditions are available upon request.

### 4.4. Structural Predictions: NOD2

Structural predictions of the human native NOD2 protein (amino-acids 219–1040) and the human R702W, G908R and N1010K variates of NOD2 proteins (amino-acids 219–1040) were obtained with the M4T server (Multiple Mapping Method with Multiple Templates, ver. 3.0) using the deduced amino-acid sequence of each protein. The predicted 3D structure of proteins was compared with the 3D-resolved structure of rabbit (*Oryctolagus cuniculus* (Oc)) NOD2 (Oc NOD2; 86 identity sequences with the human native NOD2 protein) [15] using the molecular visualization system PyMOL (The PyMOL Molecular Graphics System, Version 2.0 Schrödinger, LLC). The topology of proteins was almost completely conserved in the 3D-predicted structures, as illustrated in Figure 3. To determine whether variates of NOD2 proteins were structurally homologous to the native protein, the protein SuperPose server v.1.0 [20] was used to measure the Root-Mean-Squared Deviation (*RMSD): the average distance between the alpha carbons atoms (the backbone atoms) of superimposed proteins. The RMSD value on the domain of amino acids 219–1040 for the hR702W, hG908R and hN1010K mutated NOD2 proteins was respectively equal to: 2.86 Å, 3.34 Å and 3.48 Å. If the RMSD is below 1.5 Å, two 3D structures or domains whose sequence alignment is over 30 per-cent can be considered as almost homologous. The most significant result is correlated with the highest number of residues aligned. Regarding NOD2 proteins, there is no structural homology between the 3D-predicted structure of the human native NOD2 protein and the human hR702W, hG908R and hN1010K mutated NOD2 proteins.

### 4.5. In Silico Predictions and Annotations

Prediction of in silico pathogenicity for missenses was performed using the CADD software (Version 1.3) with calculation of the CADD Phred score [21]. A variant was predicted to be pathogenic if the CADD Phred score was above 20. Predictive tools commonly used for variant annotation including SIFT [22] and PolyPhen2 [23] were also used.

## Figures and Tables

**Table 1:** Comparison of N1010K variation and known variations associated with CD: R702W and G098R. Indicators of in silico prediction of the deleterious effects of *NOD2* variations. CADDPhred: global potential deleterious effect, SIFT: protein potential deleterious effect, PolyPhen2: protein domain potential deleterious effect, physico-chemical gap: physico-chemical gap between the 2 AA (Grantham score), modelisation gap: modelisation mid-gap between α-carbons.

	R702W	G908R	L1007fs	N1010K
ExAC MAF in Non-Finnish CEU	0.04307	0.01187	0.02319	0
GnomAD MAF	0.02355	0.007589	0.01520	0
Kaviar MAF	0.2409	0.009595	0.01279	0
CADD Phred	24.6	29.8	35.0	22.6
SIFT	0.01	0.01	N/A	0
PolyPhen2	0.72	0.986	N/A	0.996
Grantham Score	101	125	N/A	94
Modelisation gap	2.87 Å	3.34 Å	N/A	3.48 Å

**Table 2:** Montreal classification for CD. L1, pure small bowel involvement; L2, pure colonic involvement; L3, small and colonic involvements; B1, nonstricturing and non-penetrating; B2, stricturing; B3, penetrating.

Patient Identification	Age at Diagnosis (y)	Location at Diagnosis	Behaviour at Diagnosis
II:2 N1010K	30/A2	L3	B2
II:6 N1010K	24/A2	L1	B3
III:2 1007fs + N1010K	8/A1	L3	B1
III:3 1007fs + N1010K	15/A1	L3	B1

## Abbreviations

The following abbreviations are used in this manuscript:

CD	Crohn’s disease
DOAJ	Directory of open access journals
GWAS	Genome wide association study
IBD	Inflammatory Bowel Disease
LD	Linear Dichroism
LRR	Leucine-Rich Repeat
MAF	Minor Allele Frequency
MDPI	Multidisciplinary Digital Publishing Institute
NLR	NOD-like receptor
PCR	Polymerase Chain Reaction
RMSD	Root-Mean-Square-Deviation
TLA	Three letter acronym
WES	Whole Exome Sequencing
